# Co-phylogeographic structure in a disease-causing parasite and its oyster host

**DOI:** 10.1017/S0031182024000611

**Published:** 2024-06

**Authors:** Elizabeth Faye Weatherup, Ryan Carnegie, Allan E. Strand, Erik E. Sotka

**Affiliations:** 1 Virgina Institute of Marine Science, William & Mary, Gloucester Point, VA, USA; 2 Department of Biology and Marine Biology, University of North Carolina Wilmington, Wilmington, North Carolina, USA; 3 College of Charleston Marine Laboratory and Department of Biology, College of Charleston, Charleston, SC, USA

**Keywords:** co-phylogeny, *Crassostrea virginica*, genetic divergence, genotype-by-sequencing, *Perkinsus marinus*

## Abstract

With the increasing affordability of next-generation sequencing technologies, genotype-by-sequencing has become a cost-effective tool for ecologists and conservation biologists to describe a species' evolutionary history. For host–parasite interactions, genotype-by-sequencing can allow the simultaneous examination of host and parasite genomes and can yield insight into co-evolutionary processes. The eastern oyster, *Crassostrea virginica*, is among the most important aquacultured species in the United States. Natural and farmed oyster populations can be heavily impacted by ‘dermo’ disease caused by an alveolate protist, *Perkinsus marinus*. Here, we used restricted site-associated DNA sequencing (RADseq) to simultaneously examine spatial population genetic structure of host and parasite. We analysed 393 single-nucleotide polymorphisms (SNPs) for *P. marinus* and 52,100 SNPs for *C. virginica* from 36 individual oysters from the Gulf of Mexico (GOM) and mid-Atlantic coastline. All analyses revealed statistically significant genetic differentiation between the GOM and mid-Atlantic coast populations for both *C. virginica* and *P. marinus*, and genetic divergence between Chesapeake Bay and the outer coast of Virginia for *C. virginica*, but not for *P. marinus.* A co-phylogenetic analysis confirmed significant coupled evolutionary change between host and parasite across large spatial scales. The strong genetic divergence between marine basins raises the possibility that oysters from either basin would not be well adapted to parasite genotypes and phenotypes from the other, which would argue for caution with regard to both oyster and parasite transfers between the Atlantic and GOM regions. More broadly, our results demonstrate the potential of RADseq to describe spatial patterns of genetic divergence consistent with coupled evolution.

## Graphical abstract

**Figure:**
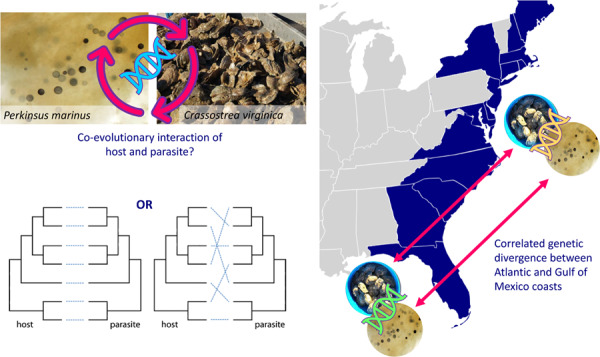

## Introduction

Microorganisms with obligate relationships with a host species commonly evolve in response to evolutionary changes in the host, by natural selection (e.g. host resistance or tolerance), or to parallel genetic divergence across biogeographic regions (Van Valen, 1973; Day, 1974; Ronquist, 1997; Page and Charleston, 1998). When host fitness is affected negatively by the presence of parasites, hosts may respond to evolutionary changes in the parasite, thus yielding co-evolutionary dynamics (Thompson, 2005). Co-evolutionary dynamics have significant implications for understanding and managing diseases in ecologically and economically important species (Coen and Bishop, 2015). Parasite tracking of host evolution, co-evolution or both yield significant patterns of coupled evolution between host and parasite genomes over space and time that can be quantified using emerging genotyping technologies (Vermeer *et al*., 2011).

Most previous efforts to study coupled evolution of host–parasite interactions using genetics utilized microsatellites or Sanger sequencing of a 1 or a few loci, which is relatively time-consuming and expensive on a per-locus basis (Ebert and Fields, 2020; Märkle *et al*., 2021). Recent advances in simultaneously amplifying single-nucleotide polymorphisms (or SNPs) using high-throughput sequencing technologies have allowed scientists the opportunity to use genotype-by-sequencing for both host and parasite genomes simultaneously. For example, Choi *et al*. (2014) used dual RNA sequencing to simultaneously look at *Brugia malayi* larvae and its mosquito host. Ansari *et al*. (2017) used a genome-wide association study (GWAS) of individuals infected with hepatitis C virus to show how SNPs in both the virus and host impacted the infection progression. Another study by Lees *et al*. (2019) also used GWAS to look at genomes of human hosts and *Streptococcus pneumoniae*, finding that host SNP variation is significant in differences in susceptibility to this bacterium. Most recently, Dexter *et al*. (2023) conducted a co-genome study of the planktonic crustacean, *Daphnia magna*, and its parasite *Pasteuria ramosa*, an endoparasitic bacterium (Dexter *et al*., 2023). They found a signal of interspecies linkage disequilibrium across multiple sets of loci demonstrating the coevolution of this host and parasite system (Dexter *et al*., 2023). Additionally, there have been several studies that have used restricted site-associated DNA sequencing (RADseq) to characterize host and parasite systems. Bracewell *et al*. (2018) used RADseq to find evidence of co-evolution and cascading speciation among 4 close interacting species, the western pine beetle, *Dendroctonus brevicomis*, the beetle's mutualistic fungi, *Ceratocystiopsis brevicomi* and *Entomocorticium* sp., and the beetle's host tree, *Pinus ponderosa* (Bracewell *et al*., 2018). Another study by Satler *et al*. (2019) was able to determine the frequent phenomena of host switching in Panamanian strangler figs and their pollinating fig wasps (Satler *et al*., 2019). A similar occurrence was found in a study by Sweet *et al*. (2020). Sweet *et al*. used whole-genome sequencing and double-digested RADseq to obtain SNPs to examine co-evolutionary patterns and host-switching accessibility between 2 species of ptarmigan birds and their associated feather lice parasites, *Lagopoecus* and *Goniodes*. They found evidence of frequent host switching of lice among different bird populations in Alaska, as well as co-evolutionary patterns between the lice and birds (Sweet *et al*., 2020).

The alveolate parasite *Perkinsus marinus* (Ph. Perkinsozoa) infects the eastern oyster *Crassostrea virginica* (Ph. Mollusca) along much of the oyster's geographic range from the Gulf of St. Lawrence, Canada to the Gulf of Mexico (GOM; Sparks, 1966). In addition to its ecological importance (Zimmerman *et al*., 1989; Smaal and Prins, 1993), *C. virginica* has been the focus of historically as well as contemporarily significant fisheries and aquaculture industries. *Perkinsus marinus* causes ‘dermo’ disease, which can lead to widespread mortality in oyster populations (Carnegie *et al*., 2021). Because of its significance, *P. marinus* has been a primary focus of regulation of aquaculture products among states of the US East Coast and the GOM, but the effectiveness of these regulations would benefit from deeper understanding of the ecological and evolutionary relationships between this host and parasite. To our knowledge, however, no previous genetic studies of *P. marinus* have genotyped SNPs nor simultaneously examined the oyster and parasite population genetics within the same individuals.

Unlike most other protozoans infecting molluscs, *P. marinus* can be cultured *in vitro*, which has made more analyses possible of the phenotypic and genetic structure of this parasite across its distribution than for other oyster parasites. In seminal early work, Bushek and Allen (1996) found that Atlantic isolates (VA and NJ) were more virulent than isolates from the GOM coast (TX and LA) in oysters collected from all 4 populations. Reece *et al*. (1997, 2001) genotyped restriction fragment length polymorphisms of *in vitro* cultures of *P. marinus*, noted the presence of diploid or multiple infections of *P. marinus* in single oysters and revealed 3 genetically distinct subdivisions among estuaries of the United States: the northeast Atlantic, southeast Atlantic and GOM (Reece *et al*., 1997, 2001). Thompson *et al*. (2011, 2014) genotyped microsatellite markers and demonstrated the presence of dimorphic loci suggesting an ancient hybridization event that persists and a fairly complex, non-equilibria pattern of spatial population structure along the Atlantic and GOM coastlines (Thompson *et al*., 2011, 2014).

Phylogeographic divergence among *C. virginica* populations is also well described. Multiple studies using a variety of mitochondrial and nuclear loci (Reeb and Avise, 1990; Hare and Avise, 1996; Varney *et al*., 2009; Thongda *et al*., 2018) have indicated divergence among and within the United States coastline of the GOM and Atlantic. These patterns reflect likely relatively short larval period (<2 weeks), entrainment of larvae within estuaries and possibly local purifying selection (Murray and Hare, 2006; Burford *et al*., 2014).

Here, we used RADseq to examine SNPs of *P. marinus* and *C. virginica* within *P. marinus*-infected oysters. Our sampling was focused on infected adults collected at 2 GOM locations (Dauphin Island, AL, and Caminada Bay, LA) and Atlantic coast locations in Virginia (Great Wicomico River, Mockhorn Bay, York River, Burtons Bay, James River and Rappahannock River). We expected a parallel phylogeographic divergence in oyster and parasite genomes from the Gulf and Atlantic coasts yielding a significant co-phylogenetic signal partitioned across the geography of this host–parasite system.

## Methods

### Collection

We genotyped 95% ethanol-preserved oyster samples from Maryland (1 location), Virginia (14 locations), Alabama (1 location) and Louisiana (2 locations), as well as an *in vitro* culture of *P. marinus* originally collected in Galveston Bay, Texas, and 1 paraffin-embedded sample initially preserved in Davidson's fixative (Shaw and Battle, 1957) (Table 1). The Virginia samples were collected during routine surveillance for oyster diseases conducted by the Carnegie lab at the Virginia Institute of Marine Science (VIMS). Samples from other states represented aquaculture industry samples submitted for disease analysis in the context of interstate shellfish commerce. For each oyster sampled, animals were cleaned, measured and shucked to expose soft tissues. Gill and mantle were extracted from each oyster and preserved in 95% ethanol for genetic analysis.

**Table 1.** Oyster populations that were positive for *P. marinus* and extracted and sequenced [AL (*n* = 3), LA (*n* = 9), VA Bay (*n* = 18), VA Eastern (*n* = 6)].

Region, state and site	Total collected	Sequenced	Lat, long	Region	Oyster type
Atlantic VA Fleet Point, Great Wicomico	3	2	37.81, −76.29	VA Chesapeake Bay	Wild
Atlantic VA Oyster	6	5	37.28, −75.90	VA Eastern Shore seaside	Wild
Atlantic VA VIMS Beach	23	14	37.25, −76.50	VA Chesapeake Bay	Wild
Atlantic VA Wachapreague	1	1	37.61, −75.66	VA Eastern Shore seaside	Wild
Atlantic VA Wreck Shoal, James River	2	1	37.06, −76.29	VA Chesapeake Bay	Wild
Atlantic VA Broad Creek, Rappahannock River	1	1	37.58, −76.30	VA Chesapeake Bay	Wild
Gulf AL Auburn University	6	3	30.25, −88.08	Dauphin Island	Farmed
Gulf LA Caminada Bay	6	5	29.24, −90.00	Grand Isle	Farmed

### Identification of oysters positive for *Perkinsus marinus*

DNA was extracted using a QIAGEN QIAamp DNA Mini Kit. Genomic DNA concentration was measured with a Nanodrop spectrophotometer to ensure the concentration was ≥100 ng *μ*L^−1^. If the concentration was >300 ng  *μ*L^−1^, then the DNA was diluted in an equal volume of Milli-Q^®^ water before polymerase chain reaction (PCR). A concentration of about 100 ng  *μ*L^−1^ was considered optimum for amplification of *P. marinus*-specific primers in each oyster sample. Samples were run on a PCR using PmarITS-70 forward primer (5′-CTT TTG YTW GAG WGT TGC GAG ATG-3′) and PmarITS-600 reverse primer (5′-CGA GTT TGC GAG TAC CTC KAG AG-3′), which target *P. marinus*. Denaturing was done at 94 °C for 30 s, annealing at 57 °C for 30 s and extension at 72 °C for 1.5 min. These steps were repeated 40 times. After PCR, to identify oysters that were positive for *P. marinus*, the PCR products were run on a 2% agarose gel at 100 volts for 30 min and stained with GelRed Nucleic Acid stain in a water bath for 25 min. This included a 1KB bp ladder at the first lane of the gel so that the desired 1000 base pair size could be visualized. Positive samples were identified and used for further analysis. The goal was to obtain 30 positive samples for each site, which was possible in some cases, but not all.

All positive samples in microcentrifuge tubes were randomly mixed before transport in case there was an error in future sequencing steps that could cause a loss in representation of an entire population. Once the microcentrifuge tubes containing the extracted DNA were thoroughly mixed, 20 *μ*L of each of the 64 samples were used in subsequent library construction.

### Library preparation

We prepared genomic libraries using the genotype-by-sequencing approach outlined in Parchman *et al*. (2012), applied to samples identified as *P. marinus*-positive. The DNA of each individual oyster was digested separately with 2 restriction enzymes, EcoRI and MseI. The digested DNA fragments were then ligated to Illumina adaptors at the MseI end and with Illumina adaptors coupled with an 8–10 bp unique barcode to the EcoRI end to allow identification of the individual *in silico*. The restriction-ligation products were then PCR-amplified in 2 separate reactions using standard Illumina primers. The final PCR products were pooled and shipped for sequencing.

Fifty-nine *P. marinus*-positive samples were sequenced in 2020 at the Tufts University Genomic Services (200–400 bp fraction; single-end sequencing on an Illumina HiSeq 2500 using SE125), 42 samples were sequenced in 2022 at the University of Texas Genomic Sequencing and Analysis Facility (300–450 bp fraction; single-end sequencing on an Illumina NovaSeq SP using SR100) and 37 of each of those samples were sequenced in both runs.

### Data filtering

Reads were aligned to the *P. marinus* genome (GCA_000006405.1) using *bwa mem* (Li and Durbin, 2010) and *samtools/bcftools* 1.9 (Danecek *et al*., 2021) using default settings. We also aligned these samples to the *C. virginica* genome (GCF_002022765.2). The median number of reads aligned to the *P. marinus* genome (0.13 M; a range of 0.10–1.01 M) was 0.37% of the median number aligned to the *C. virginica* genome (34 M; a range of 3.52–66.89 M). This yielded a ratio of 265 *C. virginica* to *P. marinus* reads per sample. Of the total number of reads per sample, a median of 0.80 and 91.30% of reads were *P. marinus* and *C. virginica*, respectively.

After alignment of 64 samples to the *P. marinus* genome, we kept 35 samples that had between 100 K and 1 M *P. marinus* reads. Another sample identified as 6837–16, from VA had 1.1 M reads, from which we randomly downsampled 500 K reads using samtools view. A final sample of 1993 TX live culture (TX-2-69, purchased from the American Type Culture Collection, ATCC) had 25 million reads that aligned with *P. marinus*; however, genotype calls showed a predominance of no calls (NAs) and consequently, it was removed. One likely reason for NAs at this step is that this culture contains multiple strains that interfere with genotype calling algorithms. For the remainder of the samples, we did not detect the presence of multiple strains nor patterns of dimorphic loci (Thompson *et al*., 2014). Thus, we were left with 36 samples (Table 1).

For *P. marinus*, we used bcftools to find all SNPs [set minor allele frequency (MAF) threshold at 0] and then a custom script to find SNPs that had at least 1 read across 50% of individuals. This yielded 772 SNPs. An analysis of allele frequencies using angsd (-doMAF 1) indicated that 393 SNPs were polymorphic between or within samples. Thus, all subsequent analyses are based on samtools-generated phred-scale genotype likelihoods of 393 polymorphic SNPs at 36 individuals. For *C. virginica*, we created genotypes from the same 36 individuals, and included SNPs with MAF > 1% and at least 1 read per sample, yielding samtools-generated genotypes at 52 100 SNPs.

### Principal component analysis

Principal component analysis (PCA) plots were generated for both organisms between collections. We used PCangsd (Meisner and Albrechtsen, 2018) on *P. marinus* genotype likelihoods and *prcomp* on *C. virginica* genotype calls within R (R Core Team, 2021). PCA is an exploratory analysis for large datasets providing more comprehensible information by grouping the data based on the individual samples' genotypic similarities.

### Analysis of molecular variance

To assess how much genetic variation is partitioned among and within groups of samples, we performed hierarchical analysis of molecular variance (AMOVA; Excoffier *et al*., 1992) using Pegas (Paradis, 2010). *P* values were generated using 1000 bootstrap replicates. For *P. marinus*, we converted the phred-scale genotype likelihoods per SNP-sample combination into probabilities that summed to 1 and then converted these to a single value ranging from 0 to 2, where 0, 1 and 2 represent the highest probability of a homozygote, heterozygote and alternative homozygote, respectively. This matrix served as an input to Pegas. For *C. virginica*, the input was genotype calls from samtools pulled using R:vcfR (Knaus and Grünwald, 2017) from a vcf file. Given the patterns found in PCA, we analysed 4 groups of samples: Alabama (*n* = 3), Louisiana (*n* = 9), Virginia Chesapeake Bay (*n* = 18) and Virginia Eastern Shore seaside (*n* = 6; see Table 1). We estimated expected heterozygosity in *P. marinus* using genotype likelihoods as implemented in *angsd*, and of genotype calls in *C. virginica* using *strataG* (Archer *et al*., 2016).

### Admixture analysis

We implemented maximum-likelihood admixture methods to infer ancestry of individuals. We used NGSadmix (Meisner and Albrechtsen, 2018) to analyse genotype likelihoods of *P. marinus* and LEA: SNMF (Frichot and François, 2015) to analyse genotype calls of *C. virginica*. Analyses were run across an *a priori* range of genetic clusters (*k* = 2–10) and replicated 10 times. Given the low number of samples, we only visualized up to *k* = 6 for both datasets. An analysis of logL changes (following Evanno *et al*., 2005) indicates that the best-fit *k* to the *P. marinus* dataset was *k* = 5. The strongest cross-validation in the SNMF *C. virginica* analysis was *k* = 6.

### Co-phylogenetic analysis

**Figure 1.**
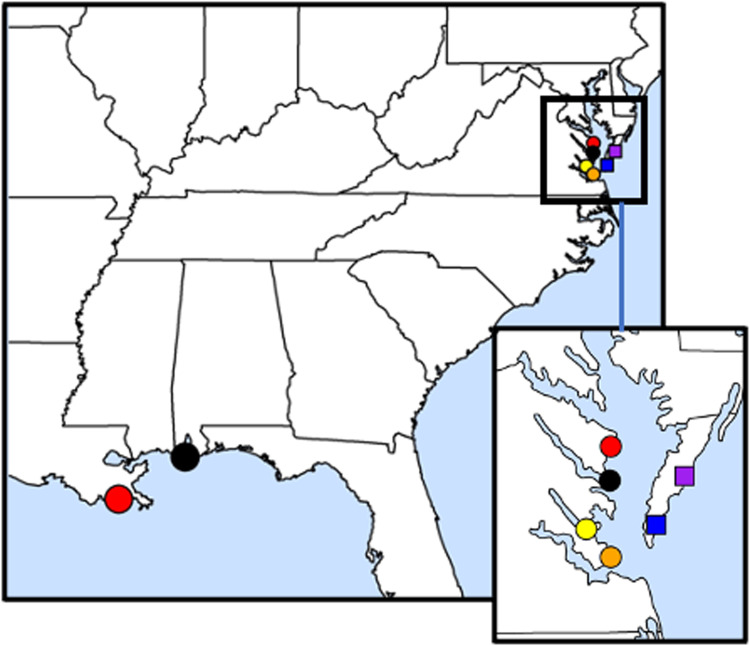
Map of geographic location of each site collection for the region of Virginia [circle, Chesapeake Bay; squares, Eastern Shore; red, Fleet Point (*n* = 2); yellow, VIMS Beach (*n* = 11); orange, Wreck Shoal (*n* = 1); black, Broad Creek (*n* = 1); purple, Wachapreague (*n* = 1); blue, Oyster (*n* = 6)] and 2 regions in the Gulf of Mexico [red = Louisiana (*n* = 5) and black = Alabama (*n* = 3)].

We visualized the co-phylogenetic structure between host and parasite using maximum-likelihood trees. We first generated fasta-formatted files of concatenated SNPs coded as IUPAC nucleotides using seqinr (Charif and Lobry, 2007). For *P. marinus*, we used PhyML implemented in SeaView (Gouy *et al*., 2010) with default parameters (GTR + 4 rate classes as a model of evolution; 100 bootstrap replicates). For *C. virginica*, we used IQTree (Trifinopoulos *et al*., 2016), which had 12766 parsimony-informative and 10028 singleton SNPs. The best-fit model of evolution was TVM + F + I + G4, allele frequencies were computed from the alignment and we used the ultrafast method to create 1000 bootstrap replicates. To statistically assess cophylogenetic signal among host and parasite, we used these trees as input to the Procrustean Approach to Co-phylogeny, or R::paco (Hutchinson *et al*., 2017). This approach assumes dependence of the parasite phylogeny on host, and assesses the probability that the observed network has more phylogenetic congruence than 1000 random instances of the interaction network (Fig. 1).

## Results

**Figure 2.**
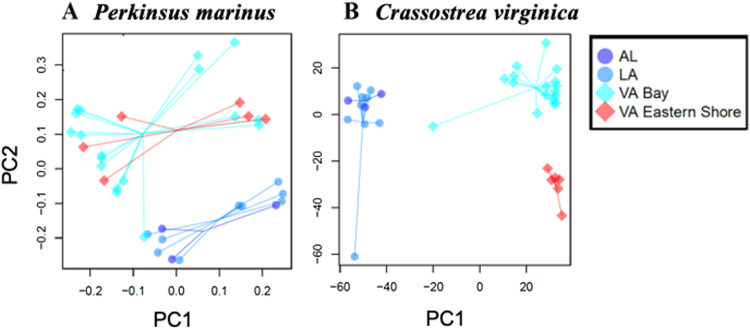
Principal components analyses of (A) *Perkinsus marinus* (393 loci, *n* = 36) from PCangsd and (B) *Crassostrea virginica* (52100 SNPs, *n* = 35) from prcomp.

**Figure 3.**
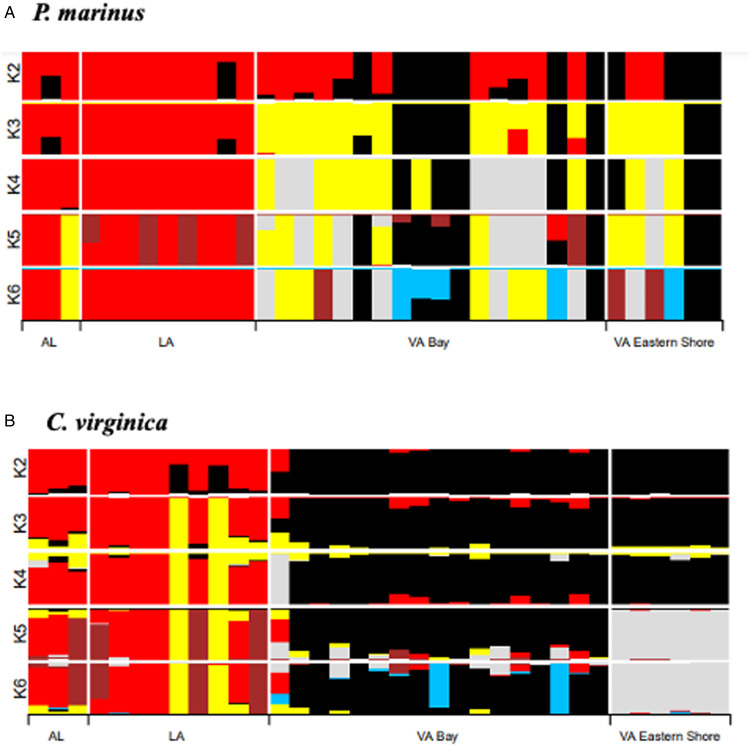
Admixture analysis of (A) *Perkinsus marinus* (393 loci, *n* = 36) using genotype likelihoods in NGSadmix and (B) *Crassostrea virginica* (52100 SNPs, *n* = 35) using genotypes in SNMF.

Strong genetic divergence between the GOM collections (LA and AL) and Virginia were revealed within the nuclear genomes of both *P. marinus* and *C. virginica* in all analyses. PCA (Fig. 2), admixture analyses (Fig. 3) and an AMOVA (Table 2) revealed strong divergence in *P. marinus* and *C. virginica* genotypes.

**Table 2.** Analysis of molecular variance (AMOVA) on *Perkinsus marinus* and *Crassostrea virginica* samples

*P. marinus*	AL	LA	VA Bay	VA Eastern
AL		0.041	0.035	0.013
LA	0.146		0.006	0.002
VA Bay	0.208	0.113		0.133
VA Eastern	0.294	0.53	0.047	
*C. virginica*	AL	LA	VA Bay	VA Eastern
AL		0.681	0.001	0.007
LA	−0.028		<0.001	<0.001
VA Bay	0.165	0.211		<0.001
VA Eastern	0.238	0.281	0.038	

Overall PhiST (i.e. across 4 populations) was 0.125 (*P* = 0.005) and 0.165 (*P* < 0.001) for *P. marinus* and *C. virginica*, respectively. Pairwise-PhiST values are shown below and above diagonals, respectively.

**Figure 4.**
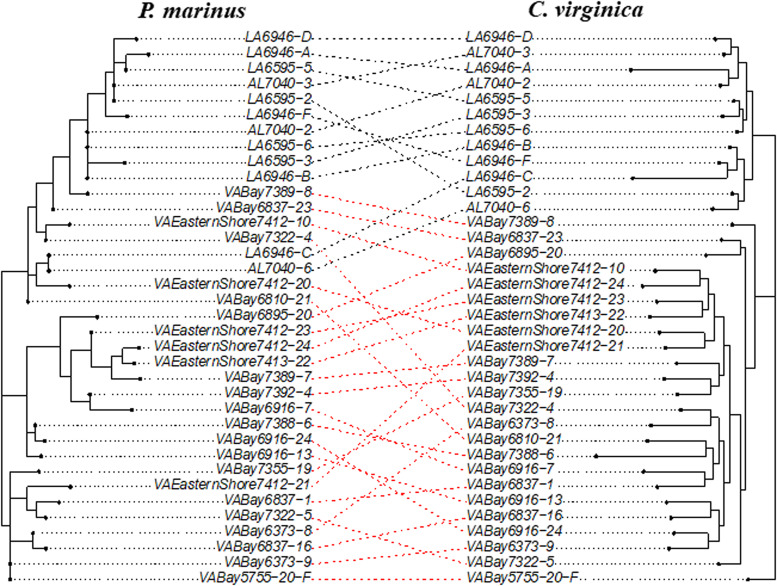
A maximum-likelihood co-phylogeny of the parasite *Perkinsus marinus* (392 bp) and host *Crassostrea virginica* (52052 bp). All nodes have 100% consensus support for *C. virginica* while all nodes have <50% support for *P. marinus* (1000 bootstrap replicates for both). Black and red dashed lines indicate GOM and VA genotypes within *Crassostrea*, respectively, and linked to *Perkinsus* genotypes.

In contrast, there was discordance between host and parasite in geographic divergence at smaller spatial scales within Virginia. *Crassostrea virginica* genotypes between Chesapeake Bay and seaside Eastern Shore groups were divergent in PCA (Fig. 2B), admixture (Fig. 3B) and AMOVA (Table 2). However, *P. marinus* genotypes were not clearly divergent in any of these analyses (Figs 2A, 3A and 4; Table 2). Regions also did not differ in expected heterozygosity for either *P. marinus* or *C. virginica* (Table 3).

**Table 3.** Expected heterozygosity (mean ± standard error across individuals) for *Crassostrea virginica* and *Perkinsus marinus*

	*n*	He *C. virginica*	s.e. *C. virginica*	He *P. marinus*	s.e. *P. marinus*
AL	3	0.294	0.155	0.015	0.046
LA	9	0.297	0.079	0.016	0.026
VA Bay	18	0.286	0.046	0.029	0.024
VA Eastern Shore	6	0.281	0.089	0.032	0.046

A co-phylogeny plot (Fig. 4) provides a view of both the phylogeographic structure and patterns of mixing between host and parasite genotypes. Overall, paco (Hutchinson *et al*., 2017) indicated a significant co-phylogenetic signal between the groups (m2xy = 0.180, *P* < 0.001, *n* = 1000). Thus, across all samples, the parasite phylogeny significantly tracked the oyster phylogeny, indicating they have undergone coupled evolutionary change, at least at the larger GOM *vs* Atlantic spatial scale. There were 2 oyster samples collected in GOM (coded LA6946-C and AL7040-6) that had *P. marinus* genotypes that were embedded within the Virginia clade (Fig. 4). This may have reflected ancestral polymorphisms from a single ancestor that have not been sorted, poor phylogenetic support in the ML tree of the *P. marinus* or more recent movement of *P. marinus* from the Atlantic to the GOM.

## Discussion

In our analyses, clear genotypic differentiation was evident between our Atlantic samples and those from the GOM, in both *P. marinus* and *C. virginica* (Figs 2 and 3; Table 2). Furthermore, we identified genotypic differentiation within *C. virginica* populations between the Chesapeake Bay and the seaside Eastern Shore in Virginia (Figs 2 and 3; Table 2). These findings support previous research on *P. marinus* (Reece *et al*., 1997, 2001; Thompson *et al*., 2014) that documented the genetic differentiation of *P. marinus* between the GOM and Mid-Atlantic regions. It is worth noting that expanding our sampling of individuals and loci could potentially unveil significant genetic structure of *P. marinus* populations within regions. Our results also align with the conclusions of Varney *et al*. (2009) and Thongda *et al*. (2018) for *C. virginica*, of genetic differentiation between and within regions. This strong genetic structure within regions likely reflects barriers to larval dispersal, local selection or both (Stauber, 1950; Narváez *et al*., 2012).

Our maximum-likelihood analysis (Fig. 4) revealed a strong signal of co-phylogeny between the parasite *P. marinus* and its oyster host. A close interaction between these 2, documented for nearly three-quarters of a century but potentially of far older origins, has resulted in an ongoing arms race, as these organisms are continually adapting to outcompete one another. This phenomenon is observed in numerous other species (Dismukes *et al*., 2022), and this methodology has the potential to uncover a multitude of evolutionary histories between hosts and parasites across diverse species.

This approach enabled us to detect genetic structure between the GOM and Atlantic *P. marinus* populations, which underscores its potential effectiveness to resolve the regional genetic diversity more finely in this pathogen. The implications for management of *P. marinus* in the context of regional shellfish aquaculture commerce are already important, even if this initial analysis was too limited to provide clear perspective on intra-regional diversity. Resource managers in the states affected by *P. marinus* parasitism of oysters are keenly interested in the question of genetic structure, because of the possibility of inadvertently creating new encounters, by pathogen introduction from distant waters through aquaculture transfers, between local oysters and *P. marinus* ‘strains’, or phenotypes, to which local oyster populations are not well adapted. The concern is that this would potentially result in a *P. marinus* epizootic of increased severity and economic destruction. Even if *P. marinus* is widely distributed and widely similar in prevalence across locations, which would argue that any aquaculture transfer of oysters with a low prevalence of *P. marinus* infections should be inconsequential against the natural backdrop of highly prevalent *P. marinus*, there is an increasing reluctance to risk introductions of *P. marinus* along with shellfish transfers because of genetic concerns. Better resolution of *P. marinus* genetic (and ideally, phenotypic) diversity could allow determination of ‘zones’ of common genotypic and phenotypic profiles within which control of *P. marinus* in transfers might be relaxed, to the benefit of reasonable aquaculture commerce and aquaculture biosecurity generally (Bushek and Allen, 1996; Reece *et al*., 1997, 2001; Carnegie *et al*., 2016). Based on our analyses, a case could already be made that divergence between Atlantic and GOM *P. marinus* populations would suggest that they should be precautionarily managed as distinct ‘zones’, between which transfers of allopatric parasite as well as oyster genotypes and phenotypes should be avoided.

This study also resulted in analysis among 393 SNPs for the parasite, despite the samples being dominated by host DNA, as it was taken from a gill and mantle sample from each oyster. The analyses were able to be examined at 52 100 SNPs for the host genome, *C. virginica*, demonstrating that with a high alignment of reads to the genome, this method can yield deep sequencing analysis, which is necessary to providing fine resolution of population genetics of species to aid in disease and risk management (Bernatchez *et al*., 2019).

### Future steps

The process of uncovering multiple loci simultaneously for both host and parasite species through RADseq and Illumina sequencing holds immense promise for future population genetic studies. However, it is important to acknowledge the limitations of our analysis, primarily stemming from the relatively small sample size and limited sample locations. To enhance the utility of this tool for co-phylogeny studies, several steps can be taken, especially regarding the parasite analysis.

First, expanding the sample size from each location to include a minimum of 30 or more *P. marinus*-positive oysters would strengthen the robustness of our analyses. This larger dataset would provide a more comprehensive understanding of the genetic structure of the parasite within and across regions, potentially allowing us to identify unique genotypes with important implications for management strategies. Another improvement involves finding a method to enrich parasite DNA, as it is significantly overshadowed by host DNA. This could be achieved by incorporating a pre-amplification step utilizing beads with attached oligonucleotides designed to specifically target the desired parasite loci, a technique supported by prior research (Shapero *et al*., 2001; Rödiger *et al*., 2014). Through the application of such amplification methods and an increased sample size, we could gain deeper insights into the genetic structure of the parasite.

## Conclusion

This study highlights genotypic divergence among regions and within regions of *P. marinus* and its host, *C. virginica* using RADseq. Unlike other studies (Bracewell *et al*., 2018; Satler *et al*., 2019; Sweet *et al*., 2020) that used RADseq, this study amplified genomes of both host and parasites from a naturally infected host, rather than a lab infection or separate sequencing. This allowed us to get an accurate and current view on the evolutionary interactions in this host and parasite system. Moreover, RADseq offers a straightforward means of assessing coupled evolutionary change within host and parasite species simultaneously.

This study could be improved with an increased sampling size of wild oysters, more sampling sites and a method to increase parasite loci. However, despite the low sampling size, this methodology provided us insight into this host parasite interaction effectively.

## Data Availability

Relevant code and datasets are found at https://github.com/esotka/WeatherupPerkinsus. FASTQ files have been uploaded to GenBank (BioSample accession SAMN39856659–95).
